# Vaccination coverage in children under one year of age and associated socioeconomic factors: maps of spatial heterogeneity

**DOI:** 10.1590/0034-7167-2022-0734

**Published:** 2023-09-18

**Authors:** Matheus Adriano Divino Pereira, Luis Henrique Arroyo, Maria Del Pilar Serrano Gallardo, Ricardo Alexandre Arcêncio, Josianne Dias Gusmão, Gabriela Gonçalves Amaral, Valéria Conceição de Oliveira, Eliete Albano de Azevedo Guimarães

**Affiliations:** I Universidade Federal de São João del-Rei. Divinópolis, Minas Gerais, Brazil; II Universidade de São Paulo. Ribeirão Preto, São Paulo, Brazil; III Universidad Autónoma de Madrid. Madrid, Spain; IV Secretaria de Estado da Saúde de Minas Gerais. Belo Horizonte, Minas Gerais, Brazil; V Universidade do Estado de Minas Gerais. Divinópolis, Minas Gerais, Brazil

**Keywords:** Vaccination, Vaccination Coverage, Child, Spatial Analysis, Ecological Studies, Vacunación, Cobertura de Vacunación, Niño, Análisis Espacial, Estudios Ecológicos, Vacinação, Cobertura Vacinal, Criança, Análise Espacial, Estudos Ecológicos

## Abstract

**Objective:**

to analyze vaccination coverage spatial distribution in children under one year old and the socioeconomic factors associated with meeting the recommended goals in Minas Gerais.

**Methods:**

an ecological study, carried out in 853 municipalities in the state. Pentavalent, poliomyelitis, meningococcal conjugate, yellow fever, rotavirus, and 10-valent pneumococcal conjugate vaccination coverage were analyzed. Scan statistics and multiple logistic regression were performed to identify spatial clusters and factors associated with meeting coverage goals.

**Results:**

spatial analysis revealed clusters with risk of low coverage for all vaccines. Number of families with per capita income of up to 1/2 wage, Minas Gerais Social Responsibility Index and percentage of the poor or extremely poor population were associated with meeting the established goals.

**Conclusions:**

the results are useful for designing interventions regarding the structuring of vaccination services and the implementation of actions to increase vaccination coverage in clusters with less propensity to vaccinate.

## RESUMO

**Objetivo:**

analisar a distribuição espacial da cobertura vacinal em menores de um ano e os fatores socioeconômicos associados ao cumprimento das metas preconizadas em Minas Gerais.

**Métodos:**

estudo ecológico, realizado nos 853 municípios do estado. Foram analisadas as coberturas vacinais da pentavalente, poliomielite, meningocócica C, febre amarela, rotavírus, e pneumocócica conjugada 10-valente. Realizou-se estatística de varredura e regressão logística múltipla para identificar agrupamentos espaciais e fatores associados ao cumprimento das metas de cobertura.

**Resultados:**

a análise espacial revelou aglomerados com risco de baixas coberturas para todas as vacinas. Número de famílias com renda per capita até 1/2 salário, Índice Mineiro de Responsabilidade Social e percentual da população pobre ou extremamente pobre se mostraram associados ao cumprimento das metas preconizadas.

**Conclusões:**

os resultados são úteis para projetar intervenções quanto à estruturação dos serviços de vacinação e implementação de ações para aumentar a cobertura vacinal nos aglomerados com menor propensão à vacinação.

## RESUMEN

**Objetivo:**

analizar la distribución espacial de las coberturas de vacunación en menores de un año y los factores socioeconómicos asociados al cumplimiento de las metas recomendadas en Minas Gerais.

**Métodos:**

estudio ecológico, realizado en 853 municipios del estado. Se analizaron las coberturas vacunales de pentavalente, poliomielitis, meningococo C, fiebre amarilla, rotavirus y neumococo conjugado decavalente. Se realizaron estadísticas de escaneo y regresión logística múltiple para identificar grupos espaciales y factores asociados con el cumplimiento de los objetivos de cobertura.

**Resultados:**

el análisis espacial reveló conglomerados con riesgo de baja cobertura para todas las vacunas. Número de familias con renta per cápita de hasta 1/2 salario, Índice de Responsabilidad Social Minas Gerais y porcentaje de población pobre o extremadamente pobre se asociaron al cumplimiento de las metas establecidas.

**Conclusiones:**

los resultados son útiles para el diseño de intervenciones en cuanto a la estructuración de los servicios de vacunación y la implementación de acciones para aumentar las coberturas de vacunación en los conglomerados con menor propensión a vacunar.

## INTRODUCTION

Vaccination coverage is a summary measure of performance used in the Brazilian National Immunization Programs (PNI) and can be monitored through administrative data or periodic vaccination coverage surveys^(1-2)^.

The Global Vaccine Action Plan 2011-2020 proposed the achievement of coverage goals for all vaccines in the Brazilian national immunization schedule by 2020. However, less than two thirds of the countries reached the proposed goal, such as the third dose of diphtheria, pertussis, and tetanus vaccine, with 66% coverage^(1)^.

In Europe, countries have shown a decline in vaccination coverage since 2016, reaching almost 14 million children without the vaccination schedule for diphtheria, pertussis, and tetanus (DPT) and measles vaccines in 2019^(3)^. In Montana, in the United States of America, less than two out of five children under two years of age had a complete schedule for childhood vaccines^(4)^.

In Brazil, since the 1990s, vaccination has shown satisfactory levels of coverage, guaranteeing access and greater equity in health^(5)^. However, as of 2016, vaccination coverage for children has declined by about 10 to 20 percentage points and, consequently, showing negative effects, such as the occurrence of epidemics, with the most recent of measles in Roraima and Amazonas^(6-8)^. It should also be noted that the rate of immunization against poliomyelitis in Brazil in 2016 was the lowest in the last 12 years (84.4%)^(9-10)^. Recently, the COVID-19 pandemic has intensified health inequalities, with low vaccination coverage for poliomyelitis and measles in socially more vulnerable and unequal municipalities^(11-12)^.

Studies carried out in Minas Gerais to analyze trends in vaccination coverage in children under two years of age, between 2014 and 2020, pointed to low vaccination coverage from 2015. Measles, mumps and rubella vaccine had coverage of less than 95% in all years analyzed. Pentavalent, Bacille Calmette-Guérin (BCG), poliomyelitis and rotavirus (LORV) vaccines were the ones that showed the greatest decreasing trend among the regions of the state^(13-14)^.

Decreases in vaccination coverage are often related to a population’s socioeconomic status and geographic conditions^(1,15-17)^, characteristics related to structural conditions and supply and access to health services in each location^(6,16,18)^ and more recently the COVID-19 pandemic^(19-20)^. Systematic monitoring of immunization coverage is an indispensable activity to know the realities in which factors ranging from the quality of management of immunization programs to political and socioeconomic factors are inserted^(15,17,21)^. This monitoring allows knowing and identifying territories that need interventions in immunization services to increase vaccine coverage^(11-12,18)^. Considering the decline in vaccination coverage in the country and among the regions of Minas Gerais, the second most populous state in Brazil^(9)^, studies are needed to support the implementation of state health policies at the regional level to increase vaccination coverage.

## OBJECTIVE

To analyze vaccination coverage spatial distribution in children under one year old and identify the socioeconomic factors associated with achieving the recommended coverage goals, in the state of Minas Gerais, in 2018.

## METHODS

### Ethical aspects

This study, in which the guidelines of Resolution 466/2012 of the Brazilian National Health Council were followed, was approved by the Research Ethics Committee. Moreover, it is derived from a master’s thesis entitled “*Análise espacial da cobertura vacinal em menores de um ano, Minas Gerais, Brasil*”, presented to the Graduate Program in Nursing at the *Universidade Federal de São João del-Rei, Centro-Oeste Dona Lindu* Campus, in 2021, and is available at: https://ufsj.edu.br/pgenf/dissertacoes_defendidas.php

### Study design, period and place

This is an ecological study carried out in the state of Minas Gerais in 2018. To elaborate the method, the STrengthening the Reporting of OBservational studies in Epidemiology recommendations were followed.

**Figure 1:**
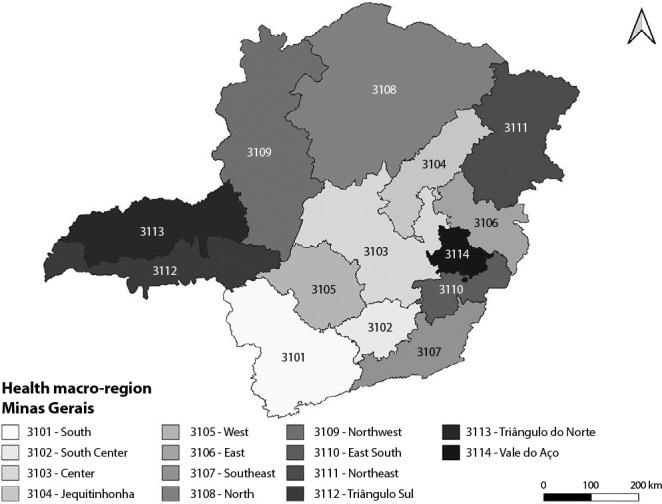
Macro-regions of the state of Minas Gerais, Brazil, 2022

The state of Minas Gerais is composed of fourteen macro-regions identified as South (3101), South Center (3102), Center (3103), Jequitinhonha (3104), West (3105), East (3106), Southeast (3107), North (3108), Northwest (3109), East South (3110), Northeast (3111), *Triângulo do Sul* (3112), *Triângulo do Norte* (3113) and *Vale do Aço* (3114) (Figure 1). These, in turn, encompass 89 health micro-regions that cover the universe of 853 municipalities^(22)^. For this study, the municipalities of the 14 macro-regions were established as territorial units of analysis.

### Study population; inclusion and exclusion criteria

The reference population for this study consisted of vaccinated children under one year old, living in the 853 municipalities of Minas Gerais. In 2017, a total of 260,959 children were registered in the Live Births Information System^(23)^, a fraction corresponding to the denominator that makes up the basis for calculating the vaccination coverage indicator for 2018.

### Study protocol

We analyzed the indicator vaccination coverage of pentavalent (DPT/Hib/HB) (dose 3), poliomyelitis (dose 3), meningococcal conjugate (MNC) (dose 2), yellow fever (YF) (single dose), LORV (dose 2) and 10-valent pneumococcal conjugate (10VPC) (dose 2) vaccines, between January and December 2018. The applied dose registration data were obtained by accessing the Unified Health System Department of Informatics electronic platform^(24)^. On this database, private network vaccines are considered for vaccination coverage calculation.

For spatial scanning analysis, the response variable was vaccination coverage, which is shown in the numerator the total doses that complete each vaccine’s scheme, and in the denominator, the number of live births in the municipality multiplied by 100. It should be noted that the Brazilian PNI established a coverage goal of 90% for LORV vaccine, 95%, for pentavalent, poliomyelitis, 10VPC and MNC vaccines, and 100%, for YF^(25)^.

With the aim of identifying the socioeconomic factors associated with achieving the goals recommended by PNI for vaccines, explanatory variables were selected from the Brazilian Institute of Geography and Statistics (IBGE - *Instituto Brasileiro de Geografia e Estatística*)^(22)^ and *Fundação João Pinheiro* ^(26)^ databases, which were included in logistic regression analyzes (Chart 1).

**Chart 1:** Explanatory variables selected for logistic regression analysis to achieve the goal of vaccination coverage in the municipalities of Minas Gerais, Brazil, 2022

Variable	Description	Source (reference year)
Urbanization rate	Ratio between the total number of people living in the urban area of the municipality and its total resident population	IBGE/*Fundação João Pinheiro* (2018)
Number of families with per capita income up to 1/2 minimum wage	Total families who were registered in the single registry, whose per capita income is equal to or less than ½ the minimum wage	Department for Information Assessment and Management - Ministry of Citizenship/*Fundação João Pinheiro* (2018)
Percentage of population aged 25 and over with a high school education	Ratio between the population aged 25 and over who completed high school and the total number of people in this age group	Atlas of Human Development in Brazil/*Fundação João Pinheiro* (2013)
Proportion of population assisted by the Family Health Strategy	Ratio between service capacity and the municipality’s total population	State Department of Health of Minas Gerais/*Fundação João Pinheiro* (2018)
Total population (adjusted estimates)	Resident population estimate	*Fundação João Pinheiro* (2018)
Budgetary effort in health activities	Share of budgetary expenditures presented in the annual rendering of accounts carried out in the subfunctions of primary care, hospital and outpatient care, prophylactic and therapeutic support, health surveillance, epidemiological surveillance and food and nutrition in total expenditures	Minas Gerais Court of Auditors/*Fundação João Pinheiro* (2018)
Index of absorption of digital technologies by municipal management	Indicator provides a summary view of each municipality’s position regarding the use of digital technologies in their management activities	Minas Gerais Court of Auditors/*Fundação João Pinheiro* (2018)
Budget effort on education activities	Participation of budget expenditures presented in the annual rendering of accounts carried out in the elementary education, high school education, professional education, higher education, child education, youth and adult education and special education subfunctions in total expenses	Minas Gerais Court of Auditors/*Fundação João Pinheiro* (2018)
Basic social protection index	Indicator composed of the existence of Comprehensive Family Protection services, Coexistence and Bond Strengthening for children aged 0 to 6 years and Coexistence and Bond Strengthening for older adults. Each service is worth 1 point, with a total of 3 points	Census of the Unified Social Assistance System/*Fundação João Pinheiro* (2018)
Percentage of families living in rural areas	Ratio between people from families living in rural areas registered in the single registry and the total population	Department for Information Assessment and Management - Ministry of Citizenship/*Fundação João Pinheiro* (2018)
Percentage of people aged 15 or over who cannot read and write and the population in this age group in the single registry	Ratio between people aged 15 or over who cannot read and write and the population in this age group registered in the municipality’s single registry	Department for Information Assessment and Management - Ministry of Citizenship/*Fundação João Pinheiro* (2018)
Percentage of population in households with a bathroom and piped water	Ratio between the population living in private households with piped water and exclusive bathroom and the total resident population	Atlas of Human Development in Brazil/*Fundação João Pinheiro* (2013)
Proportion of hospital admissions for conditions sensitive to primary care	Ratio between the number of hospital admissions for conditions sensitive to primary care and the total number of hospital admissions (Ordinance 221 of April 17, 2008)	Unified Health System Hospital Information System/*Fundação João Pinheiro* (2018)
Proportion of live births whose mothers had seven or more prenatal consultations	Ratio between the number of live births whose mothers had 7 or more prenatal consultations and the total number of live births	State Department of Health of Minas Gerais/*Fundação João Pinheiro* (2018)
20% richest/40% poorest ratio	Measure of existing degree of inequality in the distribution of individuals according to per capita household income. It compares the average per capita income of individuals belonging to the richest quintile with individuals in the poorest two quintiles	Atlas of Human Development in Brazil/*Fundação João Pinheiro* (2013)
Per capita income	Ratio between the sum of income of all individuals living in permanent private households and the total number of these individuals	Atlas of Human Development in Brazil/*Fundação João Pinheiro* (2013)
Minas Gerais Social Responsibility Index	Weighted average of the subindices referring to ten dimensions: education, health, income and employment, public safety, environment, sanitation and housing, culture, sport, tourism and leisure, social assistance and municipal finance	*Fundação João Pinheiro* (2018)
Percentage of poor or extremely poor population in the single registry in relation to the municipality’s total population	Ratio between poor or extremely poor populations registered in the single registry and the municipality’s total population	Department for Information Assessment and Management - Ministry of Citizenship/*Fundação João Pinheir*o (2018)

### Analysis of results, and statistics

Initially, data were analyzed using Microsoft Excel, version 2016, in which it was possible to calculate vaccination coverage^(25)^.

To verify the existence of clusters based on the vaccination coverage indicator, spatial scanning analysis was used, using SaTScan 9.6, supported by the discrete Poisson model^(27)^. Scan statistic operates with scanning several circular search rays through the analyzed territory, i.e., the municipalities of Minas Gerais. To define the size of these analytical circles, the maximum size of the search radius is defined, which, for the present analysis, was considered the radius of 50% of the exposed population (goal population of the analyzed vaccines). Each cluster was statistically analyzed by the log-likelihood ratio test, and its statistical significance was assessed using Monte Carlo hypothesis tests^(28)^.

Calculations of estimates for relative risk (RR) were carried out for each of the clusters identified in spatial scanning analysis. From the respective ratio, it is possible to remove the population effect, which can trigger a distortion of the analytical findings. Thus, analysis considers a variable that indicates vaccination coverage in a given cluster (group of municipalities), associating it with a given population of the respective location. Thus, a cluster’s RR is the quotient between this coverage observed in the cluster and the vaccination coverage in other municipalities in the state of Minas Gerais that do not belong to the identified cluster^(29)^. For the purposes of classification and interpretation of results in this study, when obtaining an RR>1, it can be considered that municipalities belonging to clusters have a greater chance of vaccinating their population, i.e., they are more likely to achieve high vaccination coverage compared to clusters with RR<1. Although RR is a measure calculated from a non-dichotomized variable, i.e., vaccination coverage, the respective interpretation (lower or higher chance of vaccination) was defined to provide a better understanding of results, given the large number of clusters and immunobiological agents analyzed in this research. Furthermore, it is important to highlight that RR with a value equal to one represents an unlikely association between the location and the chance of being vaccinated or not.

To prepare the choropleth maps with the results of the respective scan analysis, the cartographic base of Minas Gerais and its respective municipalities was used, obtained free of charge on the IBGE website and prepared using ArcGIS 10.8.

Considering the objective of identifying the factors associated with achieving vaccination coverage goals recommended by the PNI for the vaccines in this study, multiple logistic regressions were conducted. For this purpose, the dependent variable was considered based on the dichotomization of the municipalities that met or failed the vaccination coverage goal for the six vaccines analyzed. Thus, considering the municipalities as units of analysis for this research, they were classified as “0”, if they failed to reach the vaccination coverage goal for each of the immunobiological agents analyzed, and “1”, if this goal had been achieved by the respective location. Explanatory variables (Chart 1) were collected from different data sources to characterize the respective municipalities analyzed.

To select the final explanatory model, the lowest Akaike Information Criterion (AIC) value of the explanatory model was considered as a criterion, considering the stepwise technique of selection of variables to be included in the final statistical model. The AIC is an important metric to verify the statistical model quality, and the lower its value, the greater the quality and simplicity of the regression model. From this analytical perspective, it is important to highlight that the final explanatory model may not have all the variables presented in Chart 1, given the process of including and eliminating variables in the model in the search for the lowest possible AIC value^(30)^.

It should be noted that the Odds Ratio (OR) calculation considers “non-compliance with the recommended vaccination goal” as a reference variable, while the outcome was “achieving the recommended vaccination goal” (classification “0” for the municipality) and the outcome was “achieving the recommended vaccination goal” (classification “1”) by the municipality.

To analyze the final adjustment of the elaborated explanatory models, the Kolmogorov-Smirnov test was performed, a non-parametric test used to analyze whether the respective model’s residuals follow a normal distribution. Another test performed was McFadden’s pseudo R-squared, which measures the goodness of fit of the estimated model. Finally, the value below the Receiver Operating Characteristic Curve (ROC curve) was calculated, which analyzes variable sensitivity/specificity in the final model to predict the analyzed outcome, i.e., determines the final model’s predictive power^(30)^. A multiple logistic regression analysis was carried out for each of the six vaccines, with the respective Confidence Interval (95% CI) and p-value of the explanatory variables being calculated.

## RESULTS

Pentavalent, poliomyelitis, MNC, LORV, FAN, LORV, and 10VPC vaccination coverage was interpreted considering the health macro-regions (n=14) that make up the study analysis units (municipalities) of Minas Gerais.

The Center, *Jequitinhonha* and *Triângulo do Sul* macro-regions did not reach the recommended coverage goals for all vaccines analyzed. It was found that nine of the 14 macro-regions in Minas Gerais had adequate coverage for pentavalent, poliomyelitis and LORV vaccines, 11, for 10VPC vaccine, and eight, for MNC. The *Triângulo do Norte* macro-region was the only one to reach the YF vaccine goal (Table 1).

**Table 1:** Vaccination coverage in children under one year of age by health macro-region in Minas Gerais, Brazil, 2018

Macro-region	Vaccination coverage (%)					
	Pentavalent	Poliomyelitis	10VPC^^*^^	LORV^†^	MNC^‡^	YF^§^
South	96.9	96.3	99.9	98.5	98.8	94.6
South Center	96.8	96.9	99.4	98.4	99.2	97.6
Center	88.9	88.7	92.0	90.0	90.0	90.3
*Jequitinhonha*	91.7	90.7	91.2	89.1	88.4	87.8
West	96.1	96.1	98.8	97.9	93.3	94.2
East	96.2	95.8	100.6	97.4	98.5	90.4
Southeast	98.8	98.6	102.6	100.7	99.4	96.1
North	96.0	95.5	100.3	97.3	97.8	89.8
Northwest	98.1	98.5	103.1	100.6	99.3	95.9
East south	98.9	99.5	102.4	100.6	100.5	95.3
Northeast	94.4	92.9	95.8	93.4	93.1	88.8
*Triângulo do Sul*	89.6	88.5	92.5	89.6	86.6	81.2
*Triângulo do Norte*	124.4	124.3	127.6	127.4	126.7	114.7
Steel valley	93.8	94.0	98.3	95.8	95.0	90.8

*Note:*

* 10VPC - 10-valent pneumococcal conjugate;

† LORV - rotavirus; ^‡^MNC - meningococcal conjugate;

§ YF - yellow fever.

Spatial scan statistic detected the presence of statistically significant clusters for pentavalent, poliomyelitis, 10VPC, LORV, MNC and YF vaccination coverage. In the Center-North region, clusters with greater territorial extension (greater number of municipalities in the same cluster) were observed that had a lower chance of vaccinating their population. Considering the pentavalent, LORV and 10VPC vaccines, their clusters with the lowest chance of vaccination were identified with a propensity to form in eastern Minas Gerais, such as in the macro-regions Northeast, *Jequitinhonha*, East, *Vale do Aço* and East South. Poliomyelitis, MNC and YF vaccines were more available in central regions, encompassing macro-regions such as Center, North and Northwest, but without excluding the other regions mentioned above.

**Figure 2:**
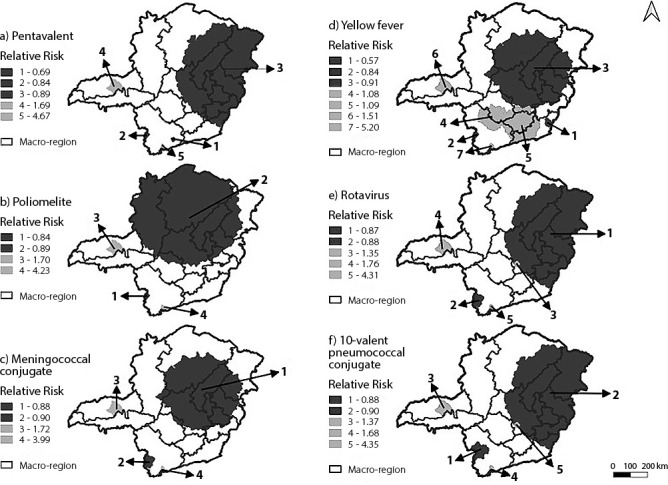
Areas of spatial clusters of vaccine coverage, referring to pentavalent, poliomyelitis, meningococcal conjugate, yellow fever, rotavirus and 10-valent pneumococcal conjugate vaccines, in children under one year old, Minas Gerais, Brazil, 2018

*Note: relative risk > 1 has a greater chance of vaccinating its population; relative risk < 1 lower chance of vaccinating the population.*

On the other hand, the *Triângulo Norte* and *Triângulo Sul* regions had clusters with a greater chance of vaccination for all immunobiological agents analyzed in the present study; however, it should be noted that these clusters had a considerably smaller territorial extension when compared to clusters with a lower chance of vaccination. It is also noteworthy that the South region was heterogeneous, as it presented clusters of lower and higher chances of vaccination together, demonstrating the complexity of the region. YF vaccine had the largest territorial dimension cluster with the greatest propensity to vaccinate the population, covering the Center-South region and four neighboring regions (Figure 2).

To identify the socioeconomic factors associated with vaccination coverage goal achievement, multiple logistic regression was conducted; for this purpose, the municipalities of Minas Gerais were dichotomized between those that met and those that did not achieve the PNI goals. YF vaccine coverage was the one with the highest number of municipalities that did not reach the recommended vaccination goal (n=292), while 10VPC had the highest number of municipalities that reached the coverage goal (n=660).

Logistic regression analysis for socioeconomic factors to achieve the vaccination coverage goal in children under one year old, in Minas Gerais, identified six associated variables, namely: number of families with per capita income up to 1/2 minimum wage; percentage of poor or extremely poor populations in the single registry in relation to the municipality’s total population; Minas Gerais Social Responsibility Index; ratio 20% richest/40% poorest; proportion of the population assisted by the Family Health Strategy; and proportion of hospital admissions for conditions sensitive to Primary Health Care. For all vaccines analyzed, it was evident that the number of families with per capita income of up to 1/2 the minimum wage, the percentage of poor or extremely poor populations in the single register in relation to the municipality’s total population and the Minas Gerais Social Responsibility Index were associated with vaccination coverage goals.

The number of families with per capita income of up to 1/2 the minimum wage was associated with all six vaccines analyzed, representing a chance of up to 0.97 (ranging between 0.96 and 0.99, when considering the 95%CI) lower for municipalities to reach indicative coverage for each new family with this income. The increase in the proportion of hospitalizations due to conditions sensitive to Primary Health Care was a factor that reduced the chances of achieving vaccination coverage for 10VPC, while the ratio between the richest 20% and the poorest 40% was related to MNC and YF, reducing by 0.92 (ranging between 0.86 and 0.99 when considering the 95%CI) the chances of achieving vaccination goals with the increase in the respective indicator, i.e., with the increase in social inequality in the municipalities (Table 2).

**Table 2:** Result of logistic regression for socioeconomic factors to achieving the goal of vaccination coverage in children under one year old, Minas Gerais, Brazil, 2018

Explanatory variable (by vaccine)	Odds Ratio	(95%) Confidence Interval	*p* value
Rotavirus			
Number of families with per capita income up to 1/2 minimum wage	0.98	0.97 - 0.99	0.002^^*^^
Percentage of poor or extremely poor populations in the single registry in relation to the municipality’s total population	1.02	1.01 - 1.04	0.015^^*^^
Minas Gerais Social Responsibility Index	36.75	1.36 - 45.27	0.032^^*^^
20% richest/40% poorest ratio	0.94	0.88 - 1.01	0.081
Proportion of population assisted by the Family Health Strategy	1.01	0.99 - 1.03	0.090
Budgetary effort in health activities	1.00	0.99 - 1.1	0.141
Index of absorption of digital technologies by municipal management	0.65	0.35 - 1.16	0.154
Meningococcal conjugate			
Number of families with per capita income up to 1/2 minimum wage	0.98	0.97 - 0.99	<0.001^^*^^
Budgetary effort in health activities	0.97	0.94 - 1.01	0.080
Proportion of population assisted by the Family Health Strategy	1.01	0.99 - 1.03	0.064
Proportion of live births whose mothers had 7 or more prenatal consultations	0.98	0.96 - 1.01	0.074
Minas Gerais Social Responsibility Index	35.72	10.77 - 43.65	0.001^^*^^
Percentage of poor or extremely poor population in the single registry in relation to the municipality’s total population	1.03	1.01 - 1.05	<0.001^^*^^
20% richest/40% poorest ratio	0.92	0.86 - 0.99	0.033^^*^^
Proportion of hospital admissions for conditions sensitive to primary care	0.99	0.98 - 1.01	0.139
10-valent pneumococcal conjugate			
Number of families with per capita income up to 1/2 minimum wage	0.98	0.97 - 0.99	0.001^^*^^
Proportion of population assisted by the Family Health Strategy	1.02	1.01 - 1.04	0.002^^*^^
Proportion of live births whose mothers had 7 or more prenatal consultations	0.98	0.97 - 1.01	0.075
Proportion of hospital admissions for conditions sensitive to primary care	0.98	0.97 - 0.99	0.038^^*^^
Budgetary effort in health activities	1.00	0.99 - 1.01	0.105
Pentavalent			
Number of families with per capita income up to 1/2 minimum wage	0.98	0.97 - 0.99	0.001^^*^^
Budgetary effort in health activities	0.97	0.94 - 1.01	0.084
urbanization rate	0.42	0.14 - 1.23	0.115
20% richest/40% poorest ratio	0.94	0.87 - 1.01	0.069
Proportion of population assisted by the Family Health Strategy	1.01	1 - 1.02	0.142
Proportion of hospital admissions for conditions sensitive to primary care	0.99	0.98 - 1	0.148
Proportion of live births whose mothers had 7 or more prenatal consultations	0.99	0.97 - 1	0.087
Minas Gerais Social Responsibility Index	63.02	31.11 - 81.70	0.017^^*^^
Percentage of poor or extremely poor population in the single registry in relation to the municipality’s total population	1.02	1.01 - 1.04	0.049^^*^^
Poliomyelitis			
Number of families with per capita income up to 1/2 minimum wage	0.98	0.97 - 0.99	<0.001^^*^^
Budgetary effort in health activities	0.96	0.94 - 1.02	0.087
Proportion of population assisted by the Family Health Strategy	1.02	1.01 - 1.03	0.024^^*^^
Proportion of hospital admissions for conditions sensitive to primary care	0.99	0.97 - 1	0.058
Yellow fever			
Number of families with per capita income up to 1/2 minimum wage	0.97	0.96 - 0.98	<0.001^^*^^
Proportion of population assisted by the Family Health Strategy	1.01	0.99 - 1.03	0.102
Minas Gerais Social Responsibility Index	33.66	26.34 - 67.90	0.002^^*^^
Percentage of poor or extremely poor population in the single registry in relation to the municipality’s total population	1.02	1.01 - 1.03	0.048^^*^^
20% richest/40% poorest ratio	0.92	0.86 - 0.98	0.017^^*^^
urbanization rate	0.42	0.15 - 1.15	0.093
Budget effort on education activities	1.00	0.99 - 1.01	0.159
Total population (adjusted estimates)	1.00	0.99 - 1.01	0.270

*Note:*

* Significant values (p<0.05)

*Model for “rotavirus”: AIC = 975.35; Kolmogorov-Smirnov test (D=0.022/p-value=0.772); McFadden’s pseudo R-squared = 0.04. Model for “meningococcal conjugate”: AIC = 925.84; Kolmogorov-Smirnov test (D=0.020/p-value=0.873); McFadden’s pseudo R-squared = 0.06. Model for “10-valent pneumococcal conjugate”: AIC = 888.83; Kolmogorov-Smirnov test (D=0.020/p-value=0.115); McFadden’s pseudo R-squared = 0.04. Model for “pentavalent”: AIC = 960.93; Kolmogorov-Smirnov test (D=0.021/p-value=0.840); McFadden’s pseudo R-squared = 0.05. Model for “poliomyelitis”: AIC = 969.82; Kolmogorov-Smirnov test (D=0.023/p-value=0.717); McFadden’s pseudo R-squared = 0.04. Model for “yellow fever”: AIC = 1041.60; Kolmogorov-Smirnov test (D=0.022/p-value=0.768); McFadden’s pseudo R-squared = 0.07.*

Among the factors that are related to an increase in the chance of reaching the goals and coverage, the Minas Gerais Social Responsibility Index was the one that represented the highest OR values, meaning a greater impact for municipalities to meet LORV, MNC, pentavalent and YF vaccine goals. However, it is important to underline that the 95%CI of this variable was extensive, meaning that the sample used in the present study does not allow an accurate representation of the studied population mean. Added to this is the proportion of people assisted by the Family Health Strategy (associated with 10VPC and poliomyelitis) and poor or extremely poor populations in the single registry (associated with LORV, MNC and pentavalent), which also showed an increase in the chances of optimal coverage for the respective vaccines that showed a significant association.

Adjustment analysis of the logistic regression models performed showed adequate values for the Kolmogorov-Smirnov test, i.e., the respective models’ residuals presented normality. When observing the results of McFadden’s pseudo R-squared, it is verified that the models presented adequate values.

Finally, when considering the area under the ROC curve for the multiple logistic regression models for LORV (ROC=0.63), MNC (ROC=0.60), 10VPC (ROC=0.60), pentavalent (ROC=0.62), poliomyelitis (ROC=0.54) and YF (ROC=0.63), it is identified that the models presented reasonable predictive power of the dependent variable, i.e., socioeconomic variables explain some conditions that lead or not the municipalities of Minas Gerais to reach the recommended PNI goal for vaccination coverage.

## DISCUSSION

Spatial distribution of pentavalent, poliomyelitis, 10VPC, LORV, MNC vaccine coverage showed differences between themselves and between the health macro-regions of Minas Gerais. It is noteworthy that the vaccination coverage of all vaccines analyzed were below the recommended goals in the Center, *Jequitinhonha* and *Triângulo do Sul* macro-regions. Socioeconomic factors were associated with the achievement of these goals, with emphasis on the number of families with per capita income of up to 1/2 minimum wage, the percentage of poor or extremely poor population in the single registry in relation to the municipality’s total population and the Minas Gerais Social Responsibility Index.

Globally, in 2020, estimated vaccine coverage was below the recommended goals for DPT and measles vaccination, with the highest annual impacts in North Africa and the Middle East, South Asia and Latin America and the Caribbean^(31)^. In Brazil, there was a downward trend in the number of immunizations for BCG, poliomyelitis and measles, mumps and rubella vaccines from 2006 to 2016^(9)^. More recently, other temporal trend studies (2011 - 2021) identified falls in vaccination coverage for poliomyelitis and measles in all Brazilian states and regions, being greater in the North and the Northeast^(11-12)^.

A study carried out in the state of Roraima that analyzed the vaccination coverage of children under one year old from 2013 to 2017 also found low coverage for the vaccines stipulated for that age group, with emphasis on the low coverage of LORV vaccine (70.4%)^(32)^. Historical series carried out in Minas Gerais identified a decreasing trend in coverage for vaccines administered to children under two years of age, with emphasis on pentavalent, LORV, BCG, hepatitis A and measles, mumps and rubella vaccines^(13-14)^. Regional variations in vaccine coverage are observed between Brazilian^(9,11,33-34)^ and Minas Gerais municipalities^(13-14)^. It is noteworthy that such municipalities comprise large territorial dimensions, with an equal emphasis on socioeconomic inequality between the different regions^(9,35)^. More specifically in the health scenario, this inequality directly affects access and care conditions, especially for the most needy or vulnerable populations^(36)^, which will consequently make it difficult to meet vaccination coverage goals^(37)^.

In Minas Gerais, the North and *Vale do Jequitinhonha* and *Mucuri* regions occupy a disadvantageous position in relation to the other regions of Minas Gerais, with the lowest percentages of social and economic indicators and municipalities with lower municipal human development indices in relation to the *Triângulo* and Center macro-regions, with excellent socioeconomic indicators^(35)^.

In this study, a polarization is noticeable between the clusters that show a lower propensity for vaccination, which are basically concentrated in the North, Northeast, East, *Jequitinhonha* and *Vale do Aço* macro-regions, and those that showed a greater propensity for the vaccinated population are present in the *Triângulo do Norte* and *Triângulo do Sul* macro-regions. Thus, Minas Gerais can be considered a representation of the Brazilian regional structure, with a poorer and less developed region in the North/Northeast and a richer and more developed region in the South^(36)^.

Low vaccination coverage is often related to geographic conditions and a population’s socioeconomic status^(1,15-16,38-39)^; structural conditions and supply and access to health services^(6,16,18,38)^; lack of knowledge of strategies recommended by the immunization program^(38,40-41)^; fake news about vaccines^(5)^; vaccine hesitancy^(5,18)^; and more recently the COVID-19 pandemic^(19-20,31)^. The latter has exacerbated pre-existing health inequalities, exposing social inequalities, discrimination and health gradients in human populations between and within countries^(12,31,42)^.

Childhood vaccination decline is heterogeneous among Brazilian municipalities, and this condition may be associated with worse indicators of human development and social inequality^(9,12,18,24,33-34)^. In this study, the number of families with per capita income of up to 1/2 the minimum wage, the Minas Gerais Social Responsibility Index and the percentage of poor or extremely poor populations included in the single registry showed an association with achieving the vaccination coverage goals.

The risk of non-compliance with vaccination coverage goals for children under one year old increases among families with per capita income of up to 1/2 the minimum wage. As families from less favored classes generally have less access to health services, it is likely that the spontaneous search for vaccination is low due to lack of infrastructure, greater distance and difficulty in accessing public services^(43-44)^. In this regard, both in the national^(45)^ and in the international scenarios^(46-48)^ it becomes evident that low level of education and low socioeconomic level are associated with vaccination below the recommended.

A study carried out to investigate disparities in vaccination coverage related to socioeconomic status, urban/rural residence and the child’s sex in 86 lowand middle-income countries identified that in 58 countries the highest levels of coverage were in urban areas and, in all countries, the poorest wealth quintile had the lowest immunization coverage^(48)^. In Brazil, research with children benefiting from *Bolsa Família* (Family Allowance), to assess vaccination coverage according to the family’s socioeconomic level and maternal characteristics, found that belonging to the richest quintile (predominantly poor sample) and maternal education ≥ 9 years were associated with higher proportions of up-to-date vaccination^(45)^.

The percentage of poor or extremely poor populations included in the single registry in relation to the municipality’s total population was positively associated with achieving vaccination coverage goals. Identifying the most vulnerable people and carrying out measures to control their social vulnerability can be an important factor in increasing vaccination coverage. A Brazilian population-based study that analyzed the impact of *Programa Bolsa Família* on child health found a positive association between receiving a benefit from the Program and greater childhood immunization coverage in low-income children^(49)^. However, a cohort carried out in São Luís and Ribeirão Preto, municipalities located in two regions with different socioeconomic conditions, identified that receiving the *Programa Bolsa Família* benefit had no influence on childhood vaccination, despite the high percentage of incomplete vaccines in São Luís (37.4%) compared to Ribeirão Preto (15.2%)^(34)^. This result may indicate that the Program’s conditionality and the monitoring of the vaccination situation are not being carried out properly, since the percentages of vaccine incompleteness in beneficiary children were high. A national longitudinal study with beneficiaries of *Programa Bolsa Família* since 2018 also found a low percentage of children with adequate vaccination, both in the first and second year of life^(45)^. Social protection and social assistance are factors to be considered as policies to strengthen vaccination. However, more effective control of program conditionalities is needed, including those related to health^(34)^.

The Minas Gerais Social Responsibility Index had the greatest impact for municipalities to meet vaccination goals in children under one year old. A study carried out in 76 countries showed that a high Human Development Index (HDI) is a predictor for greater commitment and implementation of vaccination actions^(50)^. When reviewing the factors that influence childhood vaccination schedule compliance in different countries, especially related to socioeconomic conditions, authors observed that countries with lower HDI, such as Mozambique, Uganda and Kenya, have lower vaccine coverage for DPT than countries with HDI higher^(51)^. Recently, Brazilian studies have identified clusters of low vaccination coverage for poliomyelitis and measles associated with worse human development indicators, social inequality and less access to the Family Health Strategy, facts aggravated by the COVID-19 pandemic^(11-12)^.

Achieving high and homogeneous vaccination coverage goals are essential for control and elimination of vaccine-preventable diseases, requiring global efforts and commitments to strengthen health systems and immunization services. Differences in childhood vaccination schedule compliance may be the result of different contexts of implementation of immunization programs, which pervade the health system characteristics, vaccination schedule complexity, records in the child’s vaccination book, supply of immunobiological agents and, especially, due to socioeconomic conditions^(12,18,45,52)^.

### Study limitations

Although this study sought to provide an overview of the correlates of achieving the recommended vaccination coverage goals, it is likely that there is regional variability within municipalities and also between other sets of clusters. The ecological character of this study is highlighted, in which the results presented here consider population clusters as the unit of analysis, making it impossible to interpret them at the individual level.

It can also be highlighted, as a limitation of this study, data quality and use of information produced in the PNI Information System, which can interfere with the actual calculations of vaccination coverage. A worrying reduction in the completeness of immunization records and vaccine coverage has been observed in Brazil, bringing the resurgence of some diseases hitherto overcome^(9)^.

### Contributions to nursing, health, or public policies

This study confers an important originality, by addressing an emerging problem of great social impact related to socioeconomic conditions and the supply of services, and by considering the state of Minas Gerais as its scenario, the second most populous in the country and the fourth in territorial extension. The results can support the implementation of priority measures carried out by health professionals, specifically nurses responsible for immunization services, to avoid the resurgence at the epidemic level of vaccine-preventable diseases already controlled, particularly in the face of a COVID-19 pandemic scenario caused by SARS-CoV-2, which further aggravates the population’s vaccination situation.

## CONCLUSIONS

Spatial analysis revealed clusters with risk of low vaccination coverage for pentavalent, poliomyelitis, 10VPC, LORV, MNC vaccines in Minas Gerais. Socioeconomic factors were associated with achieving vaccination coverage goals. However, the reasonable values of the area under the ROC curve (ranging from 0.54 to 0.63) show that there are other variables or conditions that need to be better analyzed in order to understand more precisely which additional factors can influence vaccination coverage in children younger than one year. Other studies should be conducted to identify other determinants for vaccine coverage.

The identification of clusters with low coverage subsidizes priority measures regarding the implementation of state health policies at the regional level, in order to increase vaccination coverage in clusters with greater spatial risk and, consequently, greater transmission of vaccine-preventable diseases.
